# Xylitol and sorbitol effects on the microbiome of saliva and plaque

**DOI:** 10.1080/20002297.2018.1536181

**Published:** 2018-10-23

**Authors:** Reisha Rafeek, Christine V. F. Carrington, Andres Gomez, Derek Harkins, Manolito Torralba, Claire Kuelbs, Jonas Addae, Ahmed Moustafa, Karen E. Nelson

**Affiliations:** a School of Dentistry, Faculty of Medical Sciences, The University of the West Indies, St. Augustine, Trinidad and Tobago; b Department of Preclinical Sciences, Faculty of Medical Sciences, The University of the West Indies, St. Augustine, Trinidad and Tobago; c Department of Genomic Medicine, J. Craig Venter Institute, La Jolla, CA, USA; d Department of Biology, The American University in Cairo, New Cairo, Egypt

**Keywords:** Caries, bacteria, saliva, plaque, microbial ecology, microbiome

## Abstract

Chewing gum containing xylitol may help prevent caries by reducing levels of mutans streptococci (MS) and lactobacilli in saliva and plaque. Very little is known about other species which are possibly beneficial to oral health. In this study, we employed high-throughput sequencing of the 16S rRNA gene to profile microbial communities of saliva and plaque following short-term consumption of xylitol and sorbitol containing chewing gum. Participants (*n* = 30) underwent a washout period and were randomly assigned to one of two groups. Each group chewed either xylitol or sorbitol gum for three weeks, before undergoing a second four-week washout period after which they switched to the alternate gum for three weeks. Analysis of samples collected before and after each intervention identified distinct plaque and saliva microbial communities that altered dependent on the order in which gum treatments were given. Neither the xylitol nor sorbitol treatments significantly affected the bacterial composition of plaque. Lactobacilli were undetected and the number of *Streptococcus mutans* sequence reads was very low and unaffected by either xylitol or sorbitol. However, sorbitol affected several other streptococcal species in saliva including increasing the abundance of *S. cristatus*, an oral commensal shown to inhibit bacteria associated with chronic periodontitis.

## Introduction

Global increases in sugar consumption have led to systemic health concerns including obesity, type 2 diabetes mellitus and oral health [1]. This has fuelled interest in sugar substitutes including polyols or non-fermentable sugars, of which the most commonly used are the nutritive sweeteners sorbitol and xylitol. Dental caries is associated with the consumption of sugars that are converted to acids by bacterial fermentation. More specifically, there is an association between caries and the presence of mutans streptococci (MS) (most notably *Streptococcus mutans* and *S. sobrinus)* and lactobacilli in saliva and plaque [2]. A systematic review of original randomized controlled trials and observational studies found that regular use of polyol-containing gum could play a role in preventing dental caries when compared to no chewing gum [3], most likely by increasing salivary flow and pH [4] and enhancing remineralization of enamel lesions [3]. Xylitol is thought to have specific anti-cariogenic properties such as the reduction of dental plaque [5] and of MS and/or lactobacilli [6–10]. Sorbitol can be fermented to a small degree whereas xylitol is not fermented by most cariogenic bacteria [11–13].

Xylitol has been approved for use in many countries, mainly as a sweetener in chewing gum. The recommended dose for caries prevention is 6–10 g/day [8]. However, some studies have found no effects of xylitol consumption on either salivary MS or lactobacilli [14,15] and a systematic review of clinical trials of xylitol – versus sorbitol-containing gum and syrup determined that the evidence to support xylitol over sorbitol was contradictory [16]. Most studies were in favour of xylitol but results were inconsistent and conflicting. Confounder risks may originate from fluoride exposure and stimulated saliva flow during trials [16].

A Cochrane review of studies using other xylitol-containing products found that over 2.5 to 3 years of use, a fluoride containing toothpaste containing 10% xylitol may reduce caries by 13% when compared to a fluoride only toothpaste [17]. The evidence was insufficient to determine whether xylitol-containing products can prevent caries in infants, older children and adults, and the conclusion was that high-quality randomized controlled trials were needed to show whether xylitol has a greater anti-cariogenic effect than sorbitol. The use of sorbitol as a control intervention in a comparison with xylitol is justified because sorbitol is the most commonly used polyol alternative to dietary sugars [18].

Many culture-based studies have focused on the effect of interventions on caries-associated bacteria [8,9,19,20], however very little is known about the effects of polyols on bacteria such as *S. sanguinis* and *S. mitis* that are thought to be beneficial to oral health [21]. There are also more than 700 bacterial species identified in the human mouth, of which an estimated 35% are uncultivated [22]. It is now possible to study complex human oral microbial populations without culturing via high-throughput sequencing of 16S rRNA genes [23,24]. Different salivary bacterial profiles have been associated with oral health and disease [25,26] and the salivary microbiome of caries-free and caries-positive subjects revealed differences in microbial community structure [27,28] with the diversity being either increased or decreased in caries compared to caries-free status depending in part on the microbiological assay used [29]. A few studies have addressed the effects of interventions on the oral microbiome utilising culture-independent approaches [14,30–34]. In this study, we used high-throughput 16S rRNA gene sequencing to investigate the impact of chewing xylitol versus sorbitol containing gums on the composition of the oral microbiota.

## Materials and methods

### Subjects

Study protocols were approved by the Ethics Committees of the UWI, St. Augustine and registered under ClinicalTrials.gov as Identifier NCT03668015 Unique Protocol ID: CRP.3.MAR14.7. Thirty healthy adult volunteers from The University of the West Indies (UWI), St. Augustine, Trinidad were enrolled in the study. To be eligible, subjects must have had at least 20 teeth, provided written informed consent and been willing to comply with study procedures. Subjects with systemic, infectious or inflammatory diseases or taking medicines, antibiotics or fluoride in the last month, habitual consumers of xylitol/sorbitol-containing products and mouth rinses, with abnormal salivary flow (<1 ml/min), pregnant, on contraceptive pills, or with abnormal dietary habits were excluded. Consent obtained at the initial visit was verified at the second visit, prior to sample collection. The subjects were examined in the dental chair after thorough medical and dental histories were recorded. The clinical examination involved examination of the soft tissues and then dental hard tissue charting for presence of decayed, missing or filled teeth. No radiographs were used. The presence of untreated dental caries or periodontitis were not used as exclusion criteria. The decayed, missing, and filled teeth for each individual at the initial visit was documented for calculation of DMFT (decayed, missing, filled teeth) score.

### Chewing gums

Xylitol gum (Epic Spearmint; 1.5 g/pellet) designated **Gum X** contains 70% xylitol in addition to gum base, natural flavours, soy lecithin, gum Arabic, titanium dioxide and carnuba wax. **Gum S** (Eclipse Spearmint), was similar except that xylitol was replaced by 63% sorbitol, and 2% maltitol and aspartame were included. Gums were packed in colour-coded containers. Codes were kept confidential from the participants and researchers who interacted with them until study completion.

### Study design

This prospective cross-over, double-blind, randomised study lasted 14 weeks (March–June 2015). Throughout, subjects were instructed not to use mouthwashes or xylitol products, to consume a normal diet, continue their usual tooth brushing and to report use of antimicrobial medications. Subjects reporting the latter were excluded.

**Figure 1.**
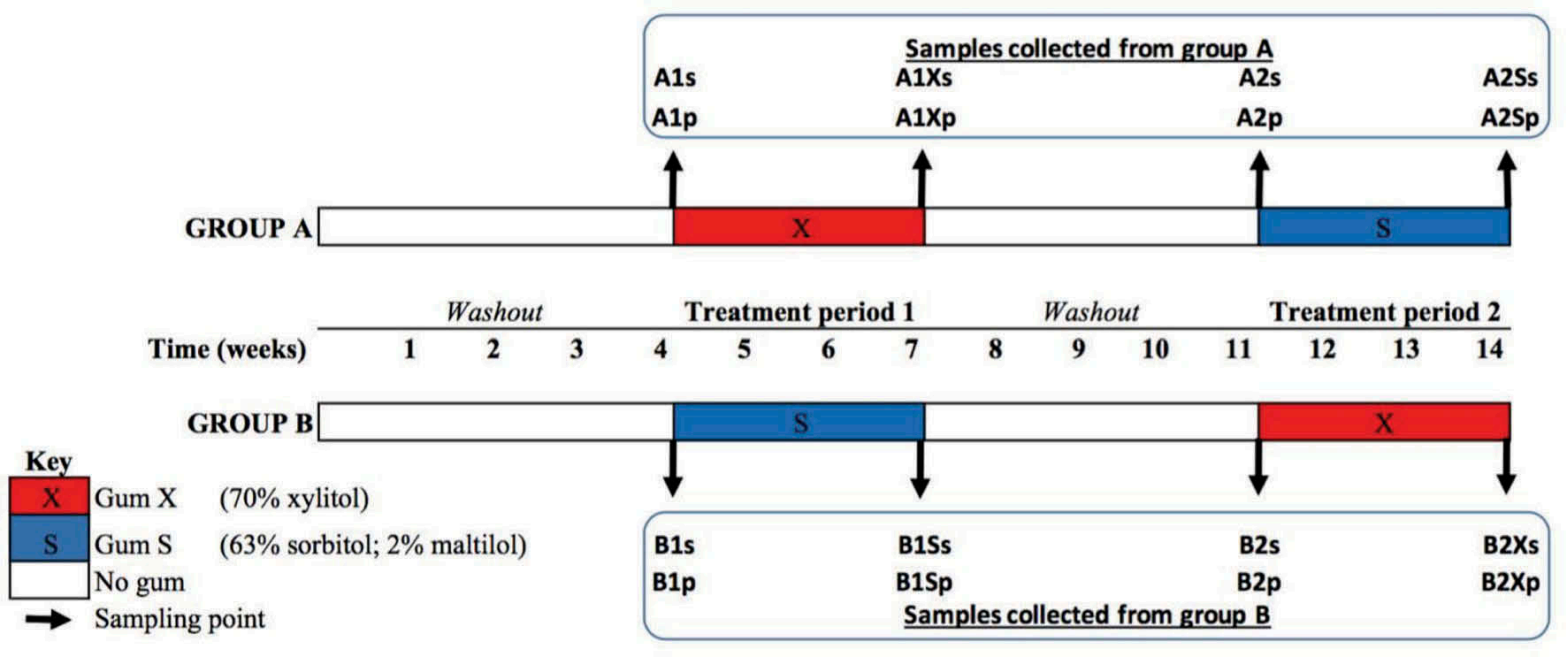
Study design. After the initial washout period, study group A was treated with gum X and group B with gum S for three weeks, followed by a second washout before treatment period 2 when group A was given gum S and group B was given gum X. Samples collected before and after each treatment period were coded according to group (A/B), the treatment period (1/2), the gum used (X/S) in the case of those samples collected at the end of a given treatment period and according to whether the sample was saliva (s) or plaque (p).

Subjects were randomly allocated to two groups, A and B (see Figure 1). Both groups entered a four-week ‘washout period’ during which no gum was chewed, followed by a three-week treatment period (treatment period 1) during which Group A used Gum X and Group B used Gum S (two gum pieces, three times daily after meals for 6 min). Both groups then underwent another four-week washout period before entering treatment period 2 during which Group A used Gum S and Group B used Gum X for three weeks.

### Sample collection

Saliva and plaque were collected from participants immediately before and after each treatment period (Figure 1). Subjects were instructed not to brush their teeth or use any other oral hygiene procedures at least 24 h before sample collection, and not to eat or drink at least 1 h before. For saliva collection, subjects chewed sterile paraffin wax and whole saliva produced was collected for 5 min in sterile tubes. Subjects were then asked to drool into the labelled sterile, conical 50 ml polypropylene collection tube with flat-top screw cap. This was repeated until 2–5 ml of saliva was collected. The saliva was transferred using sterile pipettes into labelled sterile 1.5 ml cryotubes and stored at −70°C until use.

Supragingival plaque was collected using a Gracey curette and as many strokes as necessary to remove all of the supragingival plaque from the buccal surfaces of two molars (#16 and #36), two premolars (#24 and #44), and two incisors (#21 and #41). The curette tip was immersed in sterile DNase-free TE buffer in a 1.5 ml centrifuge tube for 4–5 s. The face of the curette was wiped on the inside edge of the collection tube and then with sterile gauze to avoid introducing buffer into the patient’s mouth when the site was immediately resampled using the same procedure. After sampling was completed, the tube was closed and shaken for 4–5 s to disperse the specimen in the fluid and immediately placed on ice in a Ziploc bag before being transferred to −70°C for storage until use. Samples were labelled by group (A/B), treatment period (1/2), gum used (X/S in the case of those samples collected at the end of a given treatment period) and type (saliva (s) or plaque (p)). Samples (*n* = 232) were then shipped on dry ice to the J. Craig Venter Institute (JCVI) USA, La Jolla campus for DNA extraction and sequencing.

#### DNA extraction

Samples were thawed at 4°C and vortexed thoroughly prior to DNA extraction from 500 μl of saliva or plaque suspension using bead beating Lysing Matrix B tubes (MPBio Inc), then lysozyme digest, phenol/chloroform isoamyl alcohol extraction and ethanol precipitation were carried out. Precipitated DNA was resuspended in 1 × TE buffer.

#### Library preparation and sequencing

DNA from each sample was quantified using a Nanodrop spectrophotmeter (Thermo Fisher Scientific, Inc, Waltham, MA). Amplicons were generated using adaptor and barcode ligated PCR primers [515F: GTGCCAGCMGCCGCGGTAA; 806–787: GGACTACHVGGGTWTCTAAT] targeting the V4 region of the 16S rDNA gene (16S) and purified using Qiaquick PCR purification kits (Qiagen, Inc) following the manufacturer’s instructions. Purified amplicons were quantified using SybrGold (Thermo Fisher Scientific, Inc, Waltham, MA), normalized to ensure equimolar quantities of each sample, and pooled in preparation for Illumina MiSEQ sequencing. The 16S library pool was sequenced using the Illumina MiSEQ dual index 2 × 250 bp V2 chemistry kit according to the manufacturer’s specifications.

#### 16S RNA sequence data processing

Sequences for each sample were binned according to corresponding dual indices and exported as individual FASTQ files using CASAVA v1.8.2 (Illumina Inc, La Jolla, CA). Sequences were processed to ensure that only quality sequences were retained, as stringent settings were kept to ensure no barcode mismatches were permitted during demultiplexing. Processed sequences were applied to the Infernal pipeline [35] for additional QC checks. Bacterial sequences were taxonomically assigned based on the Genomic-based 16S ribosomal RNA Database (GRD; http://metasystems.riken.jp/grd/), which includes all sequences in the Human Oral Microbiome Database (HOMD) and allows for detection of potentially novel or specific sequences to the current study.

### Statistical analyses

Distribution by age, sex and DMFT score for subjects in groups A and B were compared using independent-samples *t*-test, Pearson’s Chi-squared test and Wilcoxon rank sum test respectively, with a cut-off of *p* value <0.05. To avoid possible sequencing errors, OTU count tables were filtered such that OTUs present in fewer than 0.1% in all samples were discarded. OTU tables were then transformed to relative abundances before community analyses were performed using the R statistical computing language [36]. Kruskal–Wallis test was used to assess statistical significance in microbial community composition across treatments. Wilcoxon test was used for pairwise comparison.

## Results

### Study group characteristics

One of 30 subjects recruited was excluded after starting antibiotics, thus a total of 29 subjects (15 female, 14 male) with a mean DMFT of 1.59 (range 0–4) were included in the final analyses. There were no significant differences between age, gender or DMFT index distributions for groups A and B (Table 1).

**Table 1.** Characteristics of study groups.

	Group A (*n* = 14)	Group B (*n* = 15)	*p* value
Age	Range: 20–27; Mean: 23.3	Range: 20–30; Mean: 23.7	0.68
DMFT	Range: 0–4; Mean 1.64	Range: 0–4; Mean 1.53	0.08
Sex	57.1% female	46.7% female	0.72

### 16S RNA sequencing and principal component analysis (PCA)

**Figure 2.**
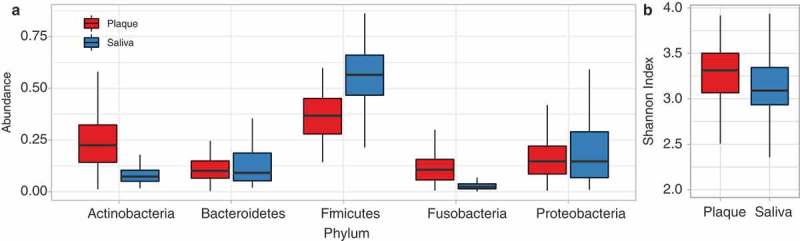
Taxonomic diversity and relative abundance in plaque compared to saliva. (a) Taxonomic abundance of bacterial phyla in plaque and saliva samples. (b) Taxonomic diversity based on Shannon Index in plaque and saliva.

Two samples (out of 232 collected (Figure 1)) were removed due to low quality/mislabelling. The remaining 230 samples yielded 13.9 million raw reads. After quality control, 4.9 million remained; each sample averaging 21,000 reads. Across all data sets, 465 OTUs were identified belonging to eight phyla, with Firmicutes accounting for the majority of reads in both plaque and saliva. Firmicutes was significantly more abundant in saliva than plaque (relative median abundance 0.56 vs 0.36; *p* value = 8.85e-25), while Actinobacteria (0.07 vs 0.22; *p* value = 8.08e-24) and Fusobacteria (0.02 vs 0.10; *p* value = 4.69e-25) were significantly less abundant in saliva than plaque (Figure 2(a)). The plaque samples were more taxonomically diverse compared to the saliva samples (*p* value <0.001) (Figure 2(b)).

**Figure 3.**
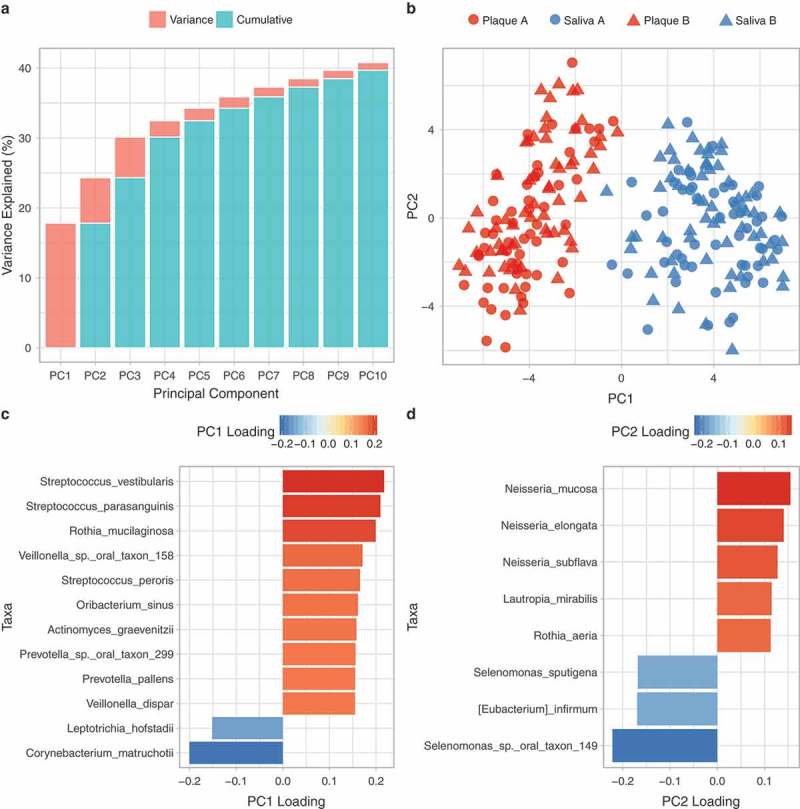
PCA of the microbial profiles of the 230 saliva and plaque samples, at the species level. (a) Individual and cumulative variance explained by the first 10 principal components. (b) Bacterial communities based on PC1 and PC2, (c) Loadings onto PC1, (d) Loadings onto PC2.

**Figure A1.**
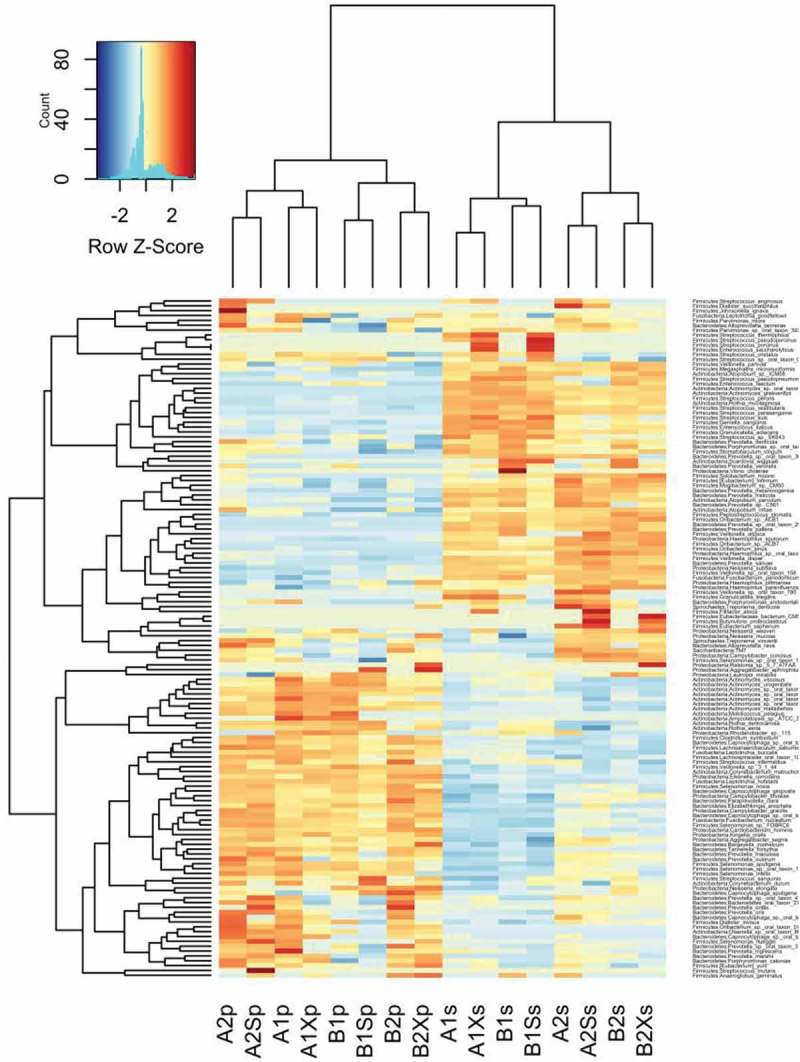
Heatmap of 16S rRNA gene abundance in samples collected from Groups A and B at each time point ordered by sample clustering horizontally and taxonomic classification vertically.

Accordingly, PCA based on the microbial profiles of the 230 samples, at the species (OTU) level (Figure 3(a,b)), indicated strong clustering primarily according to whether the sample was saliva or plaque (see also Figure A1), with most variance explained by species within the phyla Firmicutes (*S. vestibularis, S. parasanguinis, Veillonella* sp. oral taxon 158, *S. peroris, Oribacterium sinus, V. dispar, Selenomonas sputigena, Eubacterium infirmum*, Se. sp oral taxon 149), Actinobacteria (*Rothia mucilaginosa, Actinomyces graevenitzii, Corynebacterium matruchotii, R. aeria*), Bacteriodetes (*Prevotella* sp. oral taxon 299, *P. pallens*), Fusobacteria (*Leptotrichia hofstadii*) and Proteobacteria (*Neisseria mucosa, N. elongate, N. subflava, Lautropia mirailis*) (Figure 3(c,d)). The differences between saliva and plaque were greater than those driven by study group A and B (Figures 3(b) and A1) indicating that random allocation to groups was not a bias.

### Analysis of variance between abundances of species and pairwise comparisons

**Figure A2.**
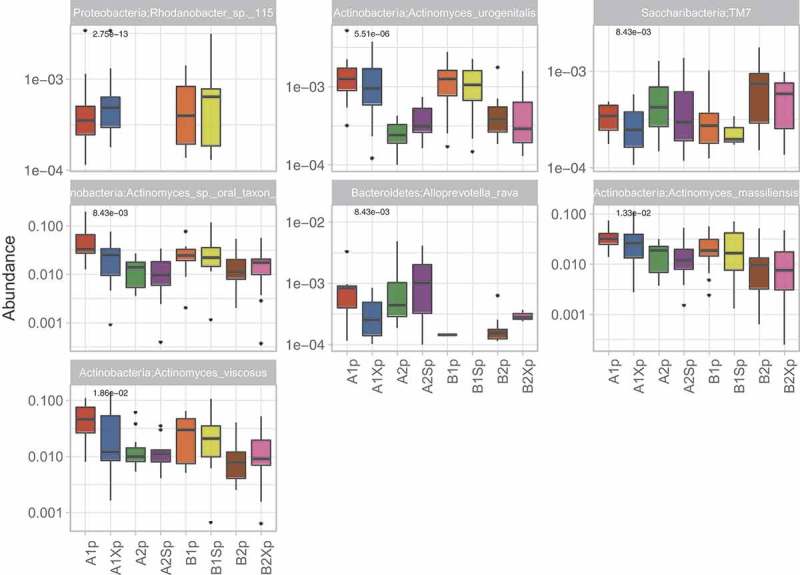
Species that showed significant differences in abundance (*p* < 0.05) among plaque samples collected from Group A and B at different time points.

**Figure A3.**
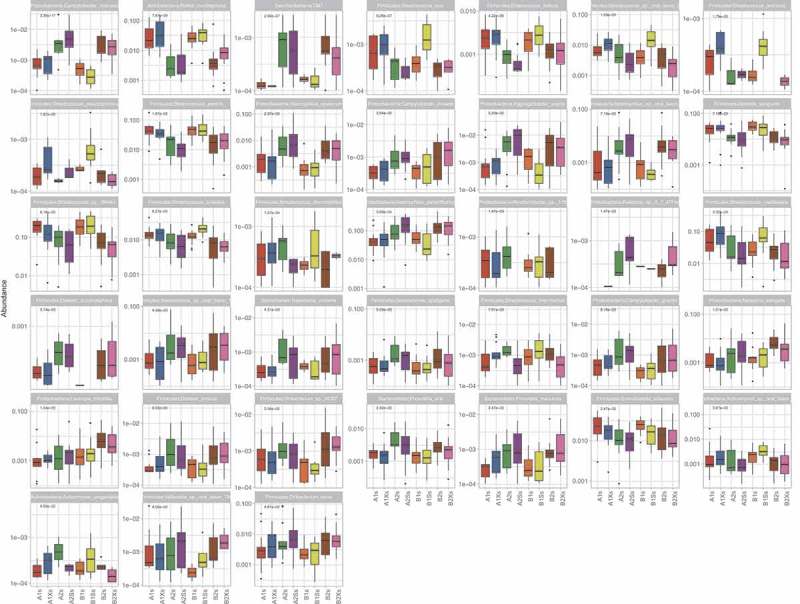
Species that showed significant differences in abundance (*p* < 0.05) among saliva samples collected from Group A and B at different time points.

**Figure A4.**
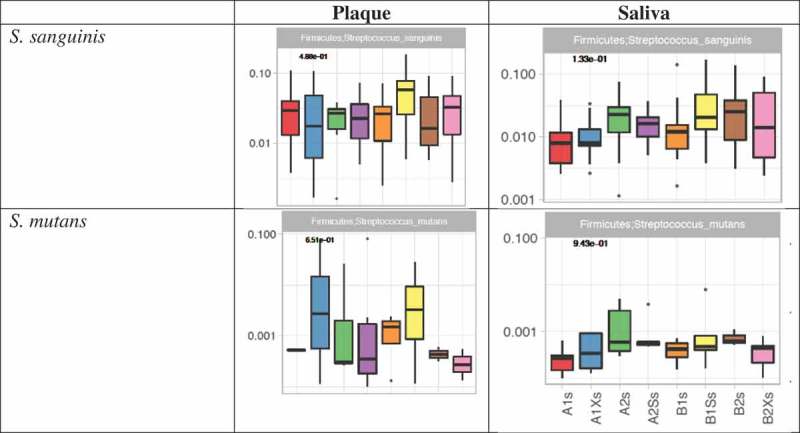
Analysis of variance of *S. sanguinis* and *S. mutans* in saliva and plaque. The *y*-axis shows relative abundance. *p* values from Kruskal–Wallis tests are shown in bold at the top left of each chart.

Kruskal–Wallis analysis of variance among samples collected from the two study groups at different time points indicated significant differences in the mean abundances of seven species belonging to four phyla (Actinobacteria (*n* = 4), Bacteroidetes (*n* = 1), Proteobacteria (*n* = 1) and Saccharibacteria (*n* = 1)) in plaque (Figure A2), and in 38 species belonging to six phyla (Actinobacteria (*n* = 3), Bacteroidetes (*n* = 2), Firmicutes (*n* = 20); Proteobacteria (*n* = 11), Saccharibacteria (*n* = 1) and Spirochaetes (*n* = 1)) in saliva (Figure A3). Previously reported caries-associated (*S. mutans, S. sobrinus, Lactobacillus*) and caries-protective (*S. mitis, S. sanguinis*) Firmicutes species were either undetected (*S. sobrinus, Lactobacillus, S. mitis)* or detected at low mean relative abundances (*S. mutans* <0.01 in plaque and <0.001 in saliva) with no significant difference across treatments *(S. mutans, S. sanguinis;*Figure A4).

Pairwise comparisons (Table A1) were made between the abundances of individual species in (i) saliva/plaque samples collected from groups A and B after the initial four-week ‘washout period’ (i.e. A1s versus B1s; A1p vs B1p), (ii) saliva/plaque from each group before and after treatment with either xylitol (A1s vs A1Xs; A1p vs A1Xp; B2s vs B2Xs; B2p vs B2Xp) or sorbitol (A2s vs A2Ss; A2p vs A2Sp; B1s vs B1Ss; B1p vs B1Sp) and (iii) between samples collected from each group at the start of their treatment 1 versus at the start of their treatment 2 (A1s vs A2s; A1p vs A2p; B1s vs B2s; B1p vs B2p).

**Figure A5.**
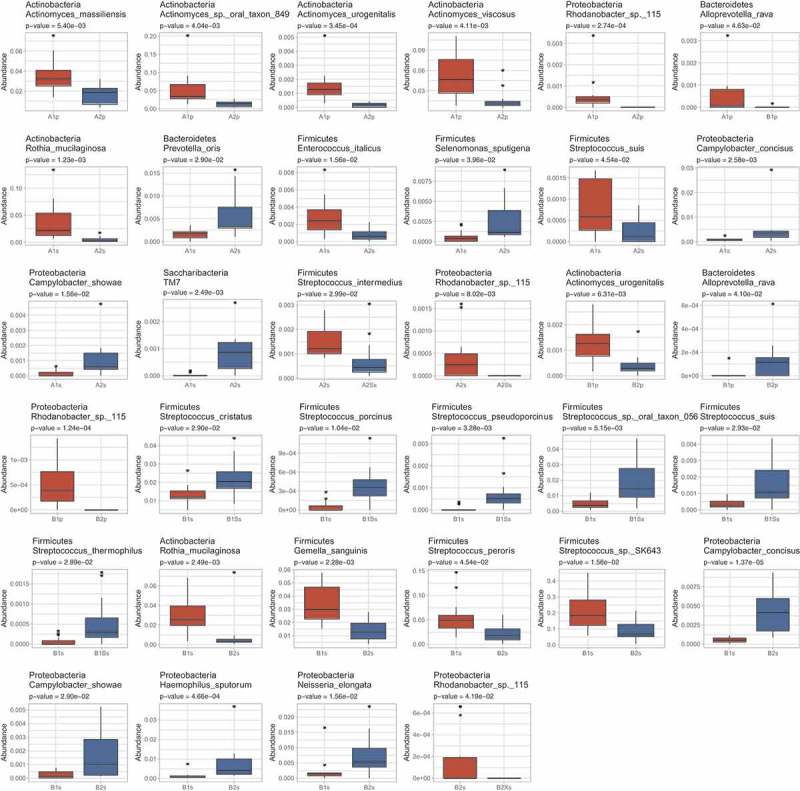
Species showing significant differences in abundance in pairwise comparisons between (i) saliva/plaque samples collected from groups A and B after the initial four-week ‘washout period’ (i.e. A1s vs B1s; A1p vs B1p), (ii) saliva/plaque from each group before and after treatment with either xylitol (A1s vs A1Xs; A1p vs A1Xp; B2s vs B2Xs; B2p vs B2Xp) or sorbitol (A2s vs A2Ss; A2p vs A2Sp; B1s vs B1Ss; B1p vs B1Sp) and (iii) between samples collected from each group at the start of their treatment 1 vs at the start of their treatment 2 (A1s vs A2s; A1p vs A2p; B1s vs B2s; B1p vs B2p).

The results (summarized in Table 2 and in Figure A5) show that before the first gum intervention there was no significant difference in the composition of group A and B saliva. Also, with the exception of one extremely low abundance species (i.e. *Alloprevotella rava*; relative mean abundance <0.001) that was slightly more abundant in group A than B (*p* value = 4.63e-02), there were no significant differences in plaque composition between groups prior to the intervention. Pairwise comparisons showed no significant change in saliva microbial composition when xylitol was given as treatment 1 (Group A). However, in group B, which received xylitol as the second treatment, there was a significant reduction in the very low abundance *Rhodanobacter* sp.115 in saliva. In contrast to the minimal effect of xylitol treatment on saliva, the three-week sorbitol treatment increased the abundance of six *Streptococcus* spp. when given as treatment 1 and decreased *S. intermedius* and *Rhodanobacter* sp. 115 when given as treatment 2. Neither xylitol nor sorbitol treatment altered plaque composition.

**Table 2.** Summary results of pairwise comparisons between samples collected from group A and B at different time points.

**(i) A1s vs B1s**: No significant difference between group A and B saliva after the initial washout period (i.e. at the start of each group’s treatment 1).			
**(ii) A1p vs B1p**: One significant difference between groups A and B plaque after the initial washout period (i.e. at the start of each group’s treatment 1).			
**Species**	**Site**	**Adjusted *p* value**	**Relative mean abundance**
*Alloprevotella rava*	Plaque	0.046	A (4.92E-04) > B (9.65E-06)
**(iii) A1s vs A1Xs**: No significant differences i.e. xylitol did not change the composition of saliva or plaque when given as treatment 1.			
**(iv) B2s vs B2Xs**: Xylitol significantly decreased one species in saliva but did not affect plaque when given as treatment 2.			
*Rhodanobacter* sp. 115	Saliva	0.042	Decreased (1.71E-04 → 0)
**(v) B1s vs B1Ss**: Sorbitol significantly increased six streptococcal species in saliva when given as treatment 1 (Group B) but did not affect plaque.			
*Streptococcus cristatus*	Saliva	0.029	Increased (0.013 → 0.022)
*Streptococcus porcinus*	Saliva	0.01	Increased (4.74E-05 → 3.72E-04)
*Streptococcus pseudoporcinus*	Saliva	0.003	Increased (5.55E-05 → 7.12E-04)
*Streptococcus* sp. oral taxon 056	Saliva	0.005	Increased (0.005 → 0.02)
*Streptococcus suis*	Saliva	0.029	Increased (3.52E-04 → 0.002)
*Streptococcus thermophilus*	Saliva	0.03	Increased (6.25E-05 → 5.08E-04)
**(vi) A2s vs A2Ss**: Sorbitol significantly decreased two species in saliva when given as treatment 2 (Group A) but did not affect plaque.			
*Streptococcus intermedius*	Saliva	0.03	Decreased (0.0015 → 7.32E-04)
*Rhodanobacter* sp. 115	Saliva	0.008	Decreased (4.31E-04 → 0)
**(vii) A1s vs A2s and A1p vs A2p**: The washout period following group A treatment with xylitol did not return saliva or plaque composition to baseline.			
*Rothia mucilaginosa*	Saliva	0.001	Higher before treatment 1 (0.04) than 2 (0.004)
*Prevotella oris*	Saliva	0.029	Lower before treatment 1 (0.002) than 2 (0.006)
*Enterococcus italicus*	Saliva	0.016	Higher before treatment 1 (0.003) than 2 (7.60E-04)
*Selenomonas sputigena*	Saliva	0.04	Lower before treatment 1 (6.03E-04) than 2 (0.003)
*Streptococcus suis*	Saliva	0.045	Higher before treatment 1 (7.96E-04) than 2 (2.11E-04)
*Campylobacter concisus*	Saliva	0.003	Lower before treatment 1 (8.20E-04) than 2 (0.0004)
*Campylobacter showae*	Saliva	0.016	Lower before treatment 1 (1.52E-04) than 2 (0.001)
*Saccharibacteria* TM7	Saliva	0.002	Lower before treatment 1 (2.83E-05) than 2 (8.38E-04)
*Actinomyces massiliensis*	Plaque	0.005	Higher before treatment 1 (0.03) than 2 (0.01)
*Actinomyces* sp. oral taxon 849	Plaque	0.004	Higher before treatment 1 (0.05) than 2 (0.01)
*Actinomyces urogenitalis*	Plaque	3.45E-04	Higher before treatment 1 (0.001) than 2 (1.76E-04)
*Actinomyces viscosus*	Plaque	0.004	Higher before treatment 1 (0.05) than 2 (0.01)
*Rhodanobacter* sp. 115	Plaque	2.74E-04	Higher before treatment 1 (5.8E-04) than 2 (0)
**(viii) B1s vs B2s and B1p vs B2p**: The washout period following group B treatment with sorbitol did not return saliva or plaque composition to baseline.			
*Rothia mucilaginosa*	Saliva	0.002	Higher before treatment 1 (0.03) than 2 (0.008)
*Gemella sanguinis*	Saliva	0.002	Higher before treatment 1 (0.03) than 2 (0.01)
*Streptococcus peroris*	Saliva	0.045	Higher before treatment 1 (0.05) than 2 (0.02)
*Streptococcus* sp. SK643	Saliva	0.016	Higher before treatment 1 (0.21) than 2 (0.09)
*Campylobacter concisus*	Saliva	1.37E-05	Lower before treatment 1 (5.14E-04) than 2 (0.004)
*Campylobacter showae*	Saliva	0.029	Lower before treatment 1 (2.41E-04) than 2 (0.002)
*Haemophilus sputorum*	Saliva	4.66E-04	Lower before treatment 1 (0.001) than 2 (0.007)
*Neisseria elongata*	Saliva	0.016	Lower before treatment 1 (0.002) than 2 (0.007)
*Actinomyces urogenitalis*	Plaque	0.006	Higher before treatment 1 (0.001) than 2 (3.9E-04)
*Alloprevotella rava*	Plaque	0.041	Lower before treatment 1 (9.65E-06) than 2 (1.12E-04)
*Rhodanobacter* sp. 115	Plaque	1.24E-04	Higher before treatment 1 (5.19E-04) than 2 (0)

Finally, pairwise comparisons demonstrated that the washout period between treatments 1 and 2 did not restore microbial composition to the pre-treatment 1 baseline (A1s vs A2s; A1p vs A2p; B1s versus B2s; B1p vs B2p) with several species at higher or lower levels in both plaque and saliva at the start of treatment.

**Figure A6.**
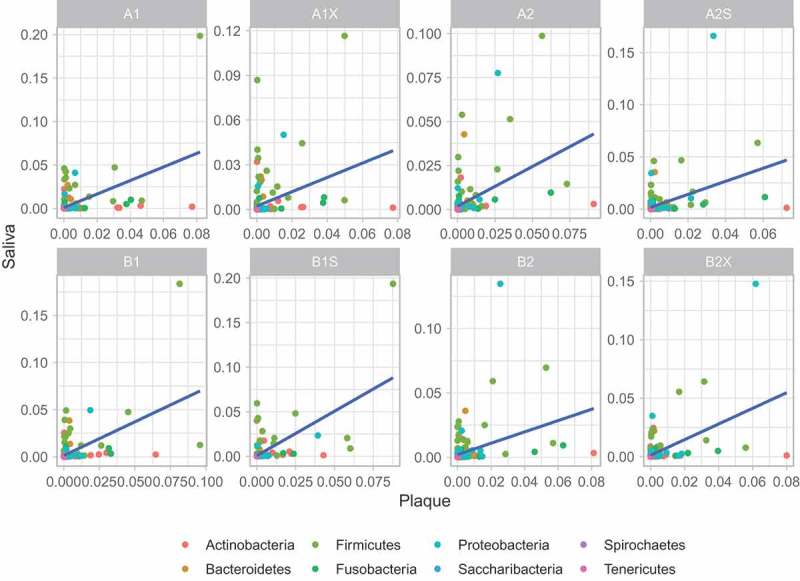
Correlation of microbiome taxa abundance between plaque and saliva samples (per condition). Each circle denotes a microbiome genus. The genera are colour-coded according to their phyla. Linear regression is indicated by the blue line with grey shade representing the confidence interval.

**Figure A7.**
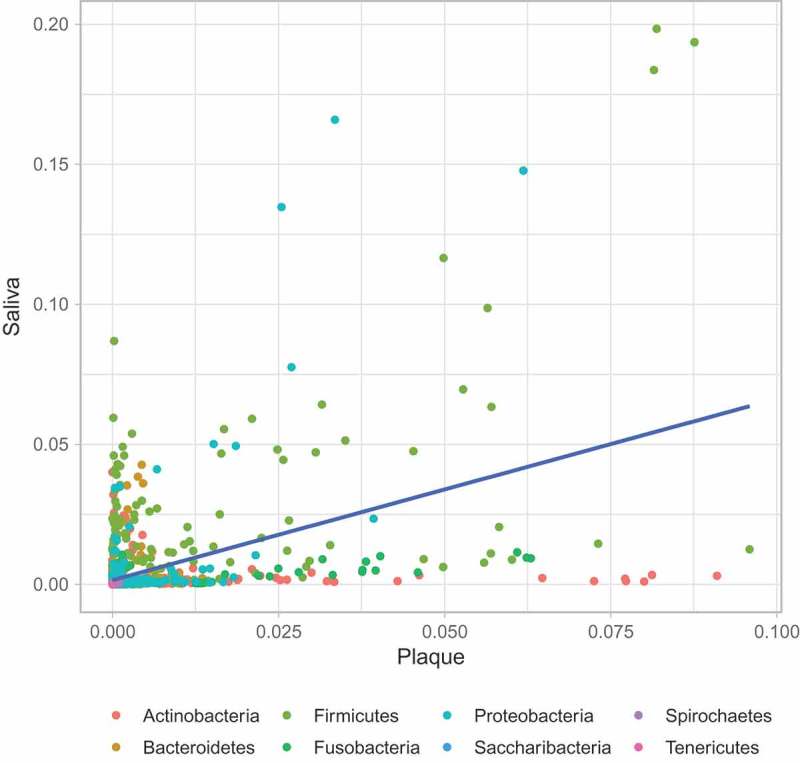
Correlation of microbiome taxa abundance between plaque and saliva samples (across all conditions). Each circle denotes a microbiome genus. The genera are colour-coded according to their phyla. Linear regression is indicated by the blue line with grey shade representing the confidence interval. *Key*Red = A1s Green = A2s Orange = B1s Brown = B2sBlue = A1Xs Purple = A2Ss Yellow = B1Ss Pink = B2Xs

Correlation analyses between supragingival plaque and saliva were performed (see Table A2 and Figures A6 and A7). This shows that albeit not strong, there is a correlation in the taxonomic abundance between saliva and plaque samples.

## Discussion

Our results revealed the distinct microbial profiles of saliva and plaque and showed that plaque microbial composition was unaffected by three-week sorbitol or xylitol treatments. In the case of saliva, the effect of sorbitol was much more pronounced than that of xylitol, which had no effect when given as treatment 1 and affected only one low abundance species when given as treatment 2. In contrast, when given as treatment 1, sorbitol increased abundance of six streptococcal species, two of which are recognised oral commensals, that is *S. cristatus* that may be beneficial by antagonizing colonization and accumulation of *Porphyromonas gingivalis* [37], a major etiologic agent contributing to chronic periodontitis, and *Streptococcus* sp. oral taxon 056. The other species (*S. porcinus, S. pseudoporcinus, S. suis* and *S. thermophilus*), although increased following sorbitol treatment, remained at very low relative abundances (<0.001). *S. pseudoporcinus* was originally isolated from the genitourinary tract of women while *S. porcinus, S. suis* and *S. thermophilus* are normally found in swine and fermented milk products respectively and may represent food contaminants. In group A, which received sorbitol as treatment 2, the aforementioned streptococcal species were unaffected but the low abundance species *S. intermedius*, part of the normal flora in the oral cavity [38] and *Rhodanobacter* sp. 115, usually found in soil [39] .

Levels of *S. mutans* were very low with no significant differences in abundance before and after either xylitol or sorbitol treatment. The latter is in contrast to previous xylitol studies involving short (three to six weeks) treatment regimens [8,18,40] and studies with treatments of two years [6,7]. The difference may be due to the fact that previous studies used culture-based approaches to detect and quantify specific bacterial species. The latter involves the use of selective media which increase sensitivity of detection for specific species but may also distort the significance of differences in abundance. For example, in the current study, *S. mutans* accounted for less than 0.006% of reads in samples taken before and after xylitol treatment; however, when samples taken from group A were cultured [9], mutans streptococci was the most commonly isolated group and there were significantly fewer colonies after xylitol than after sorbitol treatment. The V4 region of the 16S gene (used in this study) has been shown to be most representative of the microbial community [41,42] and able to capture *S. mutans* [43,44] while reducing the level of spurious OTUs and error rates. The very low abundance of *S. mutans* in our cohort may therefore be a consequence of our participants all being adults with very good oral hygiene. This may also account for the failure to detect other caries-associated species (e.g. *S. sobrinus* and lactobacilli) which some culture-based studies found to be reduced by short-term xylitol treatment [8,18,40]. Nonetheless, follow up studies to target specific oral pathogens can be conducted using pathogen-specific 16S or primers that target specific virulence factors.

The unexpected impact of the order in which xylitol and sorbitol treatments were given may be related to the fact that the four-week washout period between treatments 1 and 2 did not return the microbial composition of either saliva or plaque to pre-treatment 1 conditions, however this may not be likely as the washout period was chosen based on results of previous studies [14,40]. Alternately, one of the limitations of this study is that the diet of the participants was not controlled during the study, other than the use of chewing gums and this may have contributed to the microbiota not returning to the pre-treatment conditions. Most of the affected species (see Table 2 vii and viii), were in very low abundance. The higher abundance affected species, all recognised as part of the normal oral microflora, included *R. mucilaginosa, Gemella sanguinis, Haemophilus sputorum* (which are all occasionally associated with infections), *S. peroris* and *S*. species 643. With the exception of *H. sputorum* which was found at higher abundances before treatment 2 than treatment 1, these species were all reduced by the second washout period.

In addition to changes in species abundance, it is possible that phenotype and relative fitness of individual strains were altered by the first treatment and influenced the response to the second. Such subtle differences might also contribute to contrasting results in previous studies. For example, there are conflicting reports about the ability of lactobacilli to ferment xylitol and the effect of xylitol treatment on their abundance [12,45–47]. Interestingly, one *in vitro* study demonstrated that some lactobacilli that were initially unable to utilise xylitol adapted to xylitol use within 15–40 days of being exposed to media containing only xylitol [13].

Although in the current study the treatments had no effect on plaque (and a limited effect on saliva), significant differences in plaque composition were detected over the course of the study. The bacterial composition of plaque did however demonstrate a much lower plasticity than that of saliva (significant changes detected in 17 species in saliva versus six in plaque). The effect of the treatments on plaque is important because it is those bacteria within the plaque biofilm that adheres to the tooth surface that would promote or protect against caries. It is possible that longer term treatments are required for xylitol and sorbitol to have a significant impact on plaque.

In conclusion, our study clearly indicated significant differences in salivary and plaque microbial communities throughout the study period, including alterations in the levels of species (*S. cristatus*) thought to be protective against periodontal diseases and others that are occasionally associated with infections. However, we found no evidence that short-term consumption of gum containing xylitol or sorbitol has an impact on previously documented caries-associated or caries-protective species. Use of a control gum with all the components identical except for the xylitol, may have helped determine whether the significant difference in abundance seen in certain species would be caused by the simple use of the gum i.e. due to mastication, increased saliva flow, slight increased pH, rather than the xylitol or sorbitol. Our study participants were all adults with tertiary level education and good oral hygiene (mean DMFT = 1.59; range 0–4), only one of whom reported habitual gum chewing prior to the study period. Children or adults from the general public would likely have attitudes, knowledge and practices that might result in significantly different baseline microbial profiles. The current study also did not consider the effect of diet on microbiome composition. Further studies using longer term treatments in different groups that explore phenotypic as well as species profiles and that include diet history would therefore be useful. This work may also be complemented with *in vitro* investigation of various concentrations of xylitol/sorbitol in a biofilm model.

## Appendix

**Table A1.** Pairwise comparisons between (i) saliva/plaque samples collected from groups A and B after the initial four-week ‘washout period’ (i.e. A1s vs B1s; A1p vs B1p), (ii) saliva/plaque from each group before and after treatment with either xylitol (A1s vs A1Xs; A1p vs A1Xp; B2s vs B2Xs; B2p vs B2Xp) or sorbitol (A2s vs A2Ss; A2p vs A2Sp; B1s vs B1Ss; B1p vs B1Sp) and (iii) between samples collected from each group at the start of their treatment 1 versus at the start of their treatment 2 (A1s vs A2s; A1p vs A2p; B1s vs B2s; B1p vs B2p).

PLAQUE				
Taxonomy	Site	Name	*P* value	Adjusted *p* value
Actinobacteria;Actinomyces_massiliensis	Plaque	A1p-A1Xp	0.328666786	0.555333536
Actinobacteria;Actinomyces_sp._oral_taxon_849	Plaque	A1p-A1Xp	0.049735521	0.149189705
Actinobacteria;Actinomyces_urogenitalis	Plaque	A1p-A1Xp	0.14774294	0.297219779
Actinobacteria;Actinomyces_viscosus	Plaque	A1p-A1Xp	0.149881296	0.297219779
Bacteroidetes;Alloprevotella_rava	Plaque	A1p-A1Xp	0.151642744	0.297219779
Proteobacteria;Rhodanobacter_sp._115	Plaque	A1p-A1Xp	0.193578818	0.351309706
Saccharibacteria;TM7	Plaque	A1p-A1Xp	0.113104665	0.251914936
**Actinobacteria;Actinomyces_massiliensis**	**Plaque**	**A1p-A2p**	**6.61E-04**	**0.005399577**
**Actinobacteria;Actinomyces_sp._oral_taxon_849**	**Plaque**	**A1p-A2p**	**3.30E-04**	**0.004044186**
**Actinobacteria;Actinomyces_urogenitalis**	**Plaque**	**A1p-A2p**	**2.11E-05**	**3.45E-04**
**Actinobacteria;Actinomyces_viscosus**	**Plaque**	**A1p-A2p**	**4.19E-04**	**0.004105014**
Bacteroidetes;Alloprevotella_rava	Plaque	A1p-A2p	0.647566221	0.926028653
**Proteobacteria;Rhodanobacter_sp._115**	**Plaque**	**A1p-A2p**	**1.12E-05**	**2.74E-04**
Saccharibacteria;TM7	Plaque	A1p-A2p	0.05307183	0.149189705
Actinobacteria;Actinomyces_massiliensis	Plaque	A1p-B1p	0.045874973	0.149189705
Actinobacteria;Actinomyces_sp._oral_taxon_849	Plaque	A1p-B1p	0.093215724	0.217503357
Actinobacteria;Actinomyces_urogenitalis	Plaque	A1p-B1p	0.7471924	0.926028653
Actinobacteria;Actinomyces_viscosus	Plaque	A1p-B1p	0.04110205	0.143857174
**Bacteroidetes;Alloprevotella_rava**	**Plaque**	**A1p-B1p**	**0.00850493**	**0.04630462**
Proteobacteria;Rhodanobacter_sp._115	Plaque	A1p-B1p	0.759952871	0.926028653
Saccharibacteria;TM7	Plaque	A1p-B1p	0.7959974	0.928663633
Actinobacteria;Actinomyces_massiliensis	Plaque	A2p-A2Sp	0.756376762	0.926028653
Actinobacteria;Actinomyces_sp._oral_taxon_849	Plaque	A2p-A2Sp	0.905062742	1
Actinobacteria;Actinomyces_urogenitalis	Plaque	A2p-A2Sp	0.092342593	0.217503357
Actinobacteria;Actinomyces_viscosus	Plaque	A2p-A2Sp	0.720314184	0.926028653
Bacteroidetes;Alloprevotella_rava	Plaque	A2p-A2Sp	0.979958253	1
Proteobacteria;Rhodanobacter_sp._115	Plaque	A2p-A2Sp	1	1
Saccharibacteria;TM7	Plaque	A2p-A2Sp	0.325503952	0.555333536
Actinobacteria;Actinomyces_massiliensis	Plaque	B1p-B1Sp	0.774840302	0.926028653
Actinobacteria;Actinomyces_sp._oral_taxon_849	Plaque	B1p-B1Sp	0.743702065	0.926028653
Actinobacteria;Actinomyces_urogenitalis	Plaque	B1p-B1Sp	0.682673808	0.926028653
Actinobacteria;Actinomyces_viscosus	Plaque	B1p-B1Sp	0.9348872	1
Bacteroidetes;Alloprevotella_rava	Plaque	B1p-B1Sp	0.35064789	0.572724887
Proteobacteria;Rhodanobacter_sp._115	Plaque	B1p-B1Sp	0.45429626	0.718081184
Saccharibacteria;TM7	Plaque	B1p-B1Sp	0.054804382	0.149189705
Actinobacteria;Actinomyces_massiliensis	Plaque	B1p-B2p	0.016431722	0.067096199
Actinobacteria;Actinomyces_sp._oral_taxon_849	Plaque	B1p-B2p	0.016431722	0.067096199
**Actinobacteria;Actinomyces_urogenitalis**	**Plaque**	**B1p-B2p**	**9.01E-04**	**0.00630748**
Actinobacteria;Actinomyces_viscosus	Plaque	B1p-B2p	0.011256008	0.055154438
**Bacteroidetes;Alloprevotella_rava**	**Plaque**	**B1p-B2p**	**0.006701628**	**0.041047469**
**Proteobacteria;Rhodanobacter_sp._115**	**Plaque**	**B1p-B2p**	**2.54E-06**	**1.24E-04**
Saccharibacteria;TM7	Plaque	B1p-B2p	0.023761631	0.089563071
Actinobacteria;Actinomyces_massiliensis	Plaque	B2p-B2Xp	0.967417298	1
Actinobacteria;Actinomyces_sp._oral_taxon_849	Plaque	B2p-B2Xp	0.512486352	0.784744727
Actinobacteria;Actinomyces_urogenitalis	Plaque	B2p-B2Xp	0.90075159	1
Actinobacteria;Actinomyces_viscosus	Plaque	B2p-B2Xp	0.53929854	0.800776619
Bacteroidetes;Alloprevotella_rava	Plaque	B2p-B2Xp	0.163829361	0.308755335
Proteobacteria;Rhodanobacter_sp._115	Plaque	B2p-B2Xp	1	1
Saccharibacteria;TM7	Plaque	B2p-B2Xp	0.066010552	0.17023774
**SALIVA**				
Actinobacteria;Actinomyces_sp._oral_taxon_849	Saliva	A1s-A1Xs	0.245630188	0.568153304
Actinobacteria;Actinomyces_urogenitalis	Saliva	A1s-A1Xs	0.07890077	0.275414312
Actinobacteria;Rothia_mucilaginosa	Saliva	A1s-A1Xs	0.602685023	0.871272914
Bacteroidetes;Prevotella_maculosa	Saliva	A1s-A1Xs	0.623379069	0.882015066
Bacteroidetes;Prevotella_oris	Saliva	A1s-A1Xs	0.73445985	0.957678039
Firmicutes;Dialister_invisus	Saliva	A1s-A1Xs	0.112724945	0.339014806
Firmicutes;Dialister_succinatiphilus	Saliva	A1s-A1Xs	0.977018424	1
Firmicutes;Enterococcus_italicus	Saliva	A1s-A1Xs	0.70061057	0.950828631
Firmicutes;Gemella_sanguinis	Saliva	A1s-A1Xs	0.73445985	0.957678039
Firmicutes;Granulicatella_adiacens	Saliva	A1s-A1Xs	0.137068495	0.368285048
Firmicutes;Oribacterium_sinus	Saliva	A1s-A1Xs	0.70061057	0.950828631
Firmicutes;Oribacterium_sp._ACB7	Saliva	A1s-A1Xs	0.910025875	1
Firmicutes;Selenomonas_sp._oral_taxon_149	Saliva	A1s-A1Xs	0.945022036	1
Firmicutes;Selenomonas_sputigena	Saliva	A1s-A1Xs	0.981456799	1
Firmicutes;Streptococcus_cristatus	Saliva	A1s-A1Xs	0.803612769	0.988409148
Firmicutes;Streptococcus_intermedius	Saliva	A1s-A1Xs	0.039481661	0.172165931
Firmicutes;Streptococcus_peroris	Saliva	A1s-A1Xs	0.482400752	0.787230675
Firmicutes;Streptococcus_porcinus	Saliva	A1s-A1Xs	0.115547329	0.339014806
Firmicutes;Streptococcus_pseudoporcinus	Saliva	A1s-A1Xs	0.033518456	0.153722573
Firmicutes;Streptococcus_sp._oral_taxon_056	Saliva	A1s-A1Xs	0.049735521	0.200449221
Firmicutes;Streptococcus_sp._SK643	Saliva	A1s-A1Xs	0.137068495	0.368285048
Firmicutes;Streptococcus_suis	Saliva	A1s-A1Xs	0.334528112	0.649521736
Firmicutes;Streptococcus_thermophilus	Saliva	A1s-A1Xs	0.320885138	0.644299985
Firmicutes;Streptococcus_vestibularis	Saliva	A1s-A1Xs	0.328666786	0.644299985
Firmicutes;Veillonella_sp._oral_taxon_780	Saliva	A1s-A1Xs	1	1
Proteobacteria;Aggregatibacter_segnis	Saliva	A1s-A1Xs	0.581326862	0.859071918
Proteobacteria;Campylobacter_concisus	Saliva	A1s-A1Xs	0.981668632	1
Proteobacteria;Campylobacter_gracilis	Saliva	A1s-A1Xs	0.806043377	0.988409148
Proteobacteria;Campylobacter_showae	Saliva	A1s-A1Xs	0.363627882	0.676841283
Proteobacteria;Haemophilus_parainfluenzae	Saliva	A1s-A1Xs	0.602685023	0.871272914
Proteobacteria;Haemophilus_sp._oral_taxon_851	Saliva	A1s-A1Xs	0.662037751	0.919761774
Proteobacteria;Haemophilus_sputorum	Saliva	A1s-A1Xs	0.540926649	0.841441454
Proteobacteria;Lautropia_mirabilis	Saliva	A1s-A1Xs	1	1
Proteobacteria;Neisseria_elongata	Saliva	A1s-A1Xs	0.871626643	1
Proteobacteria;Ralstonia_sp._5_7_47FAA	Saliva	A1s-A1Xs	0.353111235	0.670911346
Proteobacteria;Rhodanobacter_sp._115	Saliva	A1s-A1Xs	0.827504996	0.997926198
Saccharibacteria;TM7	Saliva	A1s-A1Xs	1	1
Spirochaetes;Treponema_vincentii	Saliva	A1s-A1Xs	0.161337629	0.412652013
Actinobacteria;Actinomyces_sp._oral_taxon_849	Saliva	A1s-A2s	1	1
Actinobacteria;Actinomyces_urogenitalis	Saliva	A1s-A2s	0.570972361	0.85681253
**Actinobacteria;Rothia_mucilaginosa**	**Saliva**	**A1s-A2s**	**1.39E-05**	**0.001228884**
Bacteroidetes;Prevotella_maculosa	Saliva	A1s-A2s	0.009442444	0.069296138
**Bacteroidetes;Prevotella_oris**	**Saliva**	**A1s-A2s**	**0.001874935**	**0.029008045**
Firmicutes;Dialister_invisus	Saliva	A1s-A2s	0.007042085	0.060425633
Firmicutes;Dialister_succinatiphilus	Saliva	A1s-A2s	0.009638936	0.069296138
**Firmicutes;Enterococcus_italicus**	**Saliva**	**A1s-A2s**	**8.81E-04**	**0.015619314**
Firmicutes;Gemella_sanguinis	Saliva	A1s-A2s	0.012467358	0.079385837
Firmicutes;Granulicatella_adiacens	Saliva	A1s-A2s	0.016754361	0.09903689
Firmicutes;Oribacterium_sinus	Saliva	A1s-A2s	0.115874127	0.339014806
Firmicutes;Oribacterium_sp._ACB7	Saliva	A1s-A2s	0.06826431	0.257349988
Firmicutes;Selenomonas_sp._oral_taxon_149	Saliva	A1s-A2s	0.012437618	0.079385837
**Firmicutes;Selenomonas_sputigena**	**Saliva**	**A1s-A2s**	**0.003278695**	**0.039642406**
Firmicutes;Streptococcus_cristatus	Saliva	A1s-A2s	0.094478994	0.31026435
Firmicutes;Streptococcus_intermedius	Saliva	A1s-A2s	0.007795875	0.060991258
Firmicutes;Streptococcus_peroris	Saliva	A1s-A2s	0.006618208	0.058681444
Firmicutes;Streptococcus_porcinus	Saliva	A1s-A2s	0.213771447	0.516938226
Firmicutes;Streptococcus_pseudoporcinus	Saliva	A1s-A2s	0.076452032	0.271149873
Firmicutes;Streptococcus_sp._oral_taxon_056	Saliva	A1s-A2s	0.325476536	0.644299985
Firmicutes;Streptococcus_sp._SK643	Saliva	A1s-A2s	0.007795875	0.060991258
**Firmicutes;Streptococcus_suis**	**Saliva**	**A1s-A2s**	**0.004191754**	**0.045392206**
Firmicutes;Streptococcus_thermophilus	Saliva	A1s-A2s	0.32193826	0.644299985
Firmicutes;Streptococcus_vestibularis	Saliva	A1s-A2s	0.115874127	0.339014806
Firmicutes;Veillonella_sp._oral_taxon_780	Saliva	A1s-A2s	0.213164932	0.516938226
Proteobacteria;Aggregatibacter_segnis	Saliva	A1s-A2s	0.010699012	0.074893087
**Proteobacteria;Campylobacter_concisus**	**Saliva**	**A1s-A2s**	**6.78E-05**	**0.00257649**
Proteobacteria;Campylobacter_gracilis	Saliva	A1s-A2s	0.012534606	0.079385837
**Proteobacteria;Campylobacter_showae**	**Saliva**	**A1s-A2s**	**7.08E-04**	**0.015619314**
Proteobacteria;Haemophilus_parainfluenzae	Saliva	A1s-A2s	0.016754361	0.09903689
Proteobacteria;Haemophilus_sp._oral_taxon_851	Saliva	A1s-A2s	0.018578709	0.10295701
Proteobacteria;Haemophilus_sputorum	Saliva	A1s-A2s	0.054254548	0.209155214
Proteobacteria;Lautropia_mirabilis	Saliva	A1s-A2s	0.677378096	0.933588464
Proteobacteria;Neisseria_elongata	Saliva	A1s-A2s	0.366755061	0.676841283
Proteobacteria;Ralstonia_sp._5_7_47FAA	Saliva	A1s-A2s	0.005667219	0.057980009
Proteobacteria;Rhodanobacter_sp._115	Saliva	A1s-A2s	0.218344898	0.517599378
**Saccharibacteria;TM7**	**Saliva**	**A1s-A2s**	**4.83E-05**	**0.002491075**
Spirochaetes;Treponema_vincentii	Saliva	A1s-A2s	0.009638936	0.069296138
Actinobacteria;Actinomyces_sp._oral_taxon_849	Saliva	A1s-B1s	0.093215724	0.31026435
Actinobacteria;Actinomyces_urogenitalis	Saliva	A1s-B1s	0.195712209	0.484757081
Actinobacteria;Rothia_mucilaginosa	Saliva	A1s-B1s	0.651618721	0.91226621
Bacteroidetes;Prevotella_maculosa	Saliva	A1s-B1s	0.217165544	0.517599378
Bacteroidetes;Prevotella_oris	Saliva	A1s-B1s	0.600426386	0.871272914
Firmicutes;Dialister_invisus	Saliva	A1s-B1s	0.843229141	0.997926198
Firmicutes;Dialister_succinatiphilus	Saliva	A1s-B1s	0.10437015	0.330505474
Firmicutes;Enterococcus_italicus	Saliva	A1s-B1s	0.450872242	0.764232591
Firmicutes;Gemella_sanguinis	Saliva	A1s-B1s	0.15830939	0.408837843
Firmicutes;Granulicatella_adiacens	Saliva	A1s-B1s	0.914475373	1
Firmicutes;Oribacterium_sinus	Saliva	A1s-B1s	0.561290794	0.848314496
Firmicutes;Oribacterium_sp._ACB7	Saliva	A1s-B1s	0.052004482	0.203429296
Firmicutes;Selenomonas_sp._oral_taxon_149	Saliva	A1s-B1s	0.844110506	0.997926198
Firmicutes;Selenomonas_sputigena	Saliva	A1s-B1s	0.775710035	0.985855076
Firmicutes;Streptococcus_cristatus	Saliva	A1s-B1s	0.561290794	0.848314496
Firmicutes;Streptococcus_intermedius	Saliva	A1s-B1s	0.532545466	0.838207657
Firmicutes;Streptococcus_peroris	Saliva	A1s-B1s	0.948623237	1
Firmicutes;Streptococcus_porcinus	Saliva	A1s-B1s	0.26853333	0.595248882
Firmicutes;Streptococcus_pseudoporcinus	Saliva	A1s-B1s	0.093810279	0.31026435
Firmicutes;Streptococcus_sp._oral_taxon_056	Saliva	A1s-B1s	0.069658643	0.257349988
Firmicutes;Streptococcus_sp._SK643	Saliva	A1s-B1s	0.948623237	1
Firmicutes;Streptococcus_suis	Saliva	A1s-B1s	0.073197952	0.265462839
Firmicutes;Streptococcus_thermophilus	Saliva	A1s-B1s	0.017606971	0.101814226
Firmicutes;Streptococcus_vestibularis	Saliva	A1s-B1s	0.045874973	0.190667855
Firmicutes;Veillonella_sp._oral_taxon_780	Saliva	A1s-B1s	0.047556398	0.194615414
Proteobacteria;Aggregatibacter_segnis	Saliva	A1s-B1s	0.498527182	0.808586771
Proteobacteria;Campylobacter_concisus	Saliva	A1s-B1s	0.122524006	0.345929986
Proteobacteria;Campylobacter_gracilis	Saliva	A1s-B1s	0.856313971	1
Proteobacteria;Campylobacter_showae	Saliva	A1s-B1s	0.342377906	0.659945818
Proteobacteria;Haemophilus_parainfluenzae	Saliva	A1s-B1s	0.376588383	0.676841283
Proteobacteria;Haemophilus_sp._oral_taxon_851	Saliva	A1s-B1s	0.525051924	0.831332213
Proteobacteria;Haemophilus_sputorum	Saliva	A1s-B1s	0.112069791	0.339014806
Proteobacteria;Lautropia_mirabilis	Saliva	A1s-B1s	0.069418531	0.257349988
Proteobacteria;Neisseria_elongata	Saliva	A1s-B1s	0.196818665	0.484757081
Proteobacteria;Ralstonia_sp._5_7_47FAA	Saliva	A1s-B1s	0.369673441	0.676841283
Proteobacteria;Rhodanobacter_sp._115	Saliva	A1s-B1s	0.663888198	0.919761774
Saccharibacteria;TM7	Saliva	A1s-B1s	0.600889362	0.871272914
Spirochaetes;Treponema_vincentii	Saliva	A1s-B1s	0.689764282	0.945759274
Actinobacteria;Actinomyces_sp._oral_taxon_849	Saliva	A2s-A2Ss	0.274830794	0.604173481
Actinobacteria;Actinomyces_urogenitalis	Saliva	A2s-A2Ss	0.774588648	0.985855076
Actinobacteria;Rothia_mucilaginosa	Saliva	A2s-A2Ss	0.829983797	0.997926198
Bacteroidetes;Prevotella_maculosa	Saliva	A2s-A2Ss	0.576576853	0.85681253
Bacteroidetes;Prevotella_oris	Saliva	A2s-A2Ss	0.429599517	0.737248204
Firmicutes;Dialister_invisus	Saliva	A2s-A2Ss	0.375386249	0.676841283
Firmicutes;Dialister_succinatiphilus	Saliva	A2s-A2Ss	0.303023564	0.644299985
Firmicutes;Enterococcus_italicus	Saliva	A2s-A2Ss	0.609290034	0.876060265
Firmicutes;Gemella_sanguinis	Saliva	A2s-A2Ss	0.720314184	0.953251607
Firmicutes;Granulicatella_adiacens	Saliva	A2s-A2Ss	0.829983797	0.997926198
Firmicutes;Oribacterium_sinus	Saliva	A2s-A2Ss	0.550177931	0.848314496
Firmicutes;Oribacterium_sp._ACB7	Saliva	A2s-A2Ss	0.279954034	0.605429049
Firmicutes;Selenomonas_sp._oral_taxon_149	Saliva	A2s-A2Ss	0.980971668	1
Firmicutes;Selenomonas_sputigena	Saliva	A2s-A2Ss	0.375386249	0.676841283
Firmicutes;Streptococcus_cristatus	Saliva	A2s-A2Ss	0.458268747	0.766663438
**Firmicutes;Streptococcus_intermedius**	**Saliva**	**A2s-A2Ss**	**0.002275168**	**0.029894271**
Firmicutes;Streptococcus_peroris	Saliva	A2s-A2Ss	0.154582293	0.403126371
Firmicutes;Streptococcus_porcinus	Saliva	A2s-A2Ss	1	1
Firmicutes;Streptococcus_pseudoporcinus	Saliva	A2s-A2Ss	0.867643127	1
Firmicutes;Streptococcus_sp._oral_taxon_056	Saliva	A2s-A2Ss	0.325476536	0.644299985
Firmicutes;Streptococcus_sp._SK643	Saliva	A2s-A2Ss	0.302163095	0.644299985
Firmicutes;Streptococcus_suis	Saliva	A2s-A2Ss	0.324157856	0.644299985
Firmicutes;Streptococcus_thermophilus	Saliva	A2s-A2Ss	0.401761873	0.711511435
Firmicutes;Streptococcus_vestibularis	Saliva	A2s-A2Ss	0.25884048	0.585569067
Firmicutes;Veillonella_sp._oral_taxon_780	Saliva	A2s-A2Ss	0.787772175	0.985855076
Proteobacteria;Aggregatibacter_segnis	Saliva	A2s-A2Ss	0.279954034	0.605429049
Proteobacteria;Campylobacter_concisus	Saliva	A2s-A2Ss	0.140752207	0.371752149
Proteobacteria;Campylobacter_gracilis	Saliva	A2s-A2Ss	0.788920769	0.985855076
Proteobacteria;Campylobacter_showae	Saliva	A2s-A2Ss	0.770897292	0.985855076
Proteobacteria;Haemophilus_parainfluenzae	Saliva	A2s-A2Ss	0.219882443	0.517599378
Proteobacteria;Haemophilus_sp._oral_taxon_851	Saliva	A2s-A2Ss	0.720314184	0.953251607
Proteobacteria;Haemophilus_sputorum	Saliva	A2s-A2Ss	0.238822532	0.557252575
Proteobacteria;Lautropia_mirabilis	Saliva	A2s-A2Ss	0.941472333	1
Proteobacteria;Neisseria_elongata	Saliva	A2s-A2Ss	0.789425305	0.985855076
Proteobacteria;Ralstonia_sp._5_7_47FAA	Saliva	A2s-A2Ss	0.827390383	0.997926198
**Proteobacteria;Rhodanobacter_sp._115**	**Saliva**	**A2s-A2Ss**	**3.01E-04**	**0.008016039**
Saccharibacteria;TM7	Saliva	A2s-A2Ss	0.48139587	0.787230675
Spirochaetes;Treponema_vincentii	Saliva	A2s-A2Ss	0.941661916	1
Actinobacteria;Actinomyces_sp._oral_taxon_849	Saliva	B1s-B1Ss	0.11597875	0.339014806
Actinobacteria;Actinomyces_urogenitalis	Saliva	B1s-B1Ss	0.097166967	0.312552435
Actinobacteria;Rothia_mucilaginosa	Saliva	B1s-B1Ss	0.412375778	0.716940895
Bacteroidetes;Prevotella_maculosa	Saliva	B1s-B1Ss	0.307364925	0.644299985
Bacteroidetes;Prevotella_oris	Saliva	B1s-B1Ss	0.950384432	1
Firmicutes;Dialister_invisus	Saliva	B1s-B1Ss	0.505967063	0.810766498
Firmicutes;Dialister_succinatiphilus	Saliva	B1s-B1Ss	0.35064789	0.670911346
Firmicutes;Enterococcus_italicus	Saliva	B1s-B1Ss	0.106445694	0.333112406
Firmicutes;Gemella_sanguinis	Saliva	B1s-B1Ss	0.324548175	0.644299985
Firmicutes;Granulicatella_adiacens	Saliva	B1s-B1Ss	0.032943642	0.153722573
Firmicutes;Oribacterium_sinus	Saliva	B1s-B1Ss	0.838134777	0.997926198
Firmicutes;Oribacterium_sp._ACB7	Saliva	B1s-B1Ss	0.616465037	0.876896791
Firmicutes;Selenomonas_sp._oral_taxon_149	Saliva	B1s-B1Ss	0.558988678	0.848314496
Firmicutes;Selenomonas_sputigena	Saliva	B1s-B1Ss	0.258256792	0.585569067
**Firmicutes;Streptococcus_cristatus**	**Saliva**	**B1s-B1Ss**	**0.00196295**	**0.029008045**
Firmicutes;Streptococcus_intermedius	Saliva	B1s-B1Ss	0.09752576	0.312552435
Firmicutes;Streptococcus_peroris	Saliva	B1s-B1Ss	0.806333778	0.988409148
**Firmicutes;Streptococcus_porcinus**	**Saliva**	**B1s-B1Ss**	**4.28E-04**	**0.010357423**
**Firmicutes;Streptococcus_pseudoporcinus**	**Saliva**	**B1s-B1Ss**	**9.87E-05**	**0.003280212**
**Firmicutes;Streptococcus_sp._oral_taxon_056**	**Saliva**	**B1s-B1Ss**	**1.74E-04**	**0.005146773**
Firmicutes;Streptococcus_sp._SK643	Saliva	B1s-B1Ss	0.967417298	1
**Firmicutes;Streptococcus_suis**	**Saliva**	**B1s-B1Ss**	**0.002096373**	**0.029349216**
**Firmicutes;Streptococcus_thermophilus**	**Saliva**	**B1s-B1Ss**	**0.002360074**	**0.029894271**
Firmicutes;Streptococcus_vestibularis	Saliva	B1s-B1Ss	0.029417889	0.140108255
Firmicutes;Veillonella_sp._oral_taxon_780	Saliva	B1s-B1Ss	0.575867202	0.85681253
Proteobacteria;Aggregatibacter_segnis	Saliva	B1s-B1Ss	0.073850564	0.265462839
Proteobacteria;Campylobacter_concisus	Saliva	B1s-B1Ss	0.038002593	0.170303502
Proteobacteria;Campylobacter_gracilis	Saliva	B1s-B1Ss	0.715600392	0.953251607
Proteobacteria;Campylobacter_showae	Saliva	B1s-B1Ss	0.451069612	0.764232591
Proteobacteria;Haemophilus_parainfluenzae	Saliva	B1s-B1Ss	0.126147401	0.345929986
Proteobacteria;Haemophilus_sp._oral_taxon_851	Saliva	B1s-B1Ss	0.42439585	0.733047378
Proteobacteria;Haemophilus_sputorum	Saliva	B1s-B1Ss	0.870194456	1
Proteobacteria;Lautropia_mirabilis	Saliva	B1s-B1Ss	0.917402105	1
Proteobacteria;Neisseria_elongata	Saliva	B1s-B1Ss	1	1
Proteobacteria;Ralstonia_sp._5_7_47FAA	Saliva	B1s-B1Ss	1	1
Proteobacteria;Rhodanobacter_sp._115	Saliva	B1s-B1Ss	0.897680214	1
Saccharibacteria;TM7	Saliva	B1s-B1Ss	0.614620907	0.876896791
Spirochaetes;Treponema_vincentii	Saliva	B1s-B1Ss	0.882112318	1
Actinobacteria;Actinomyces_sp._oral_taxon_849	Saliva	B1s-B2s	0.126147401	0.345929986
Actinobacteria;Actinomyces_urogenitalis	Saliva	B1s-B2s	0.463214051	0.770093361
**Actinobacteria;Rothia_mucilaginosa**	**Saliva**	**B1s-B2s**	**5.62E-05**	**0.002491075**
Bacteroidetes;Prevotella_maculosa	Saliva	B1s-B2s	0.180285455	0.456723152
Bacteroidetes;Prevotella_oris	Saliva	B1s-B2s	0.092947853	0.31026435
Firmicutes;Dialister_invisus	Saliva	B1s-B2s	0.01991441	0.107122963
Firmicutes;Dialister_succinatiphilus	Saliva	B1s-B2s	0.025238903	0.126670722
Firmicutes;Enterococcus_italicus	Saliva	B1s-B2s	0.020135895	0.107122963
**Firmicutes;Gemella_sanguinis**	**Saliva**	**B1s-B2s**	**3.43E-05**	**0.002282437**
Firmicutes;Granulicatella_adiacens	Saliva	B1s-B2s	0.007543545	0.060991258
Firmicutes;Oribacterium_sinus	Saliva	B1s-B2s	0.018553726	0.10295701
Firmicutes;Oribacterium_sp._ACB7	Saliva	B1s-B2s	0.259763722	0.585569067
Firmicutes;Selenomonas_sp._oral_taxon_149	Saliva	B1s-B2s	0.051213548	0.203325427
Firmicutes;Selenomonas_sputigena	Saliva	B1s-B2s	0.027907646	0.137470998
Firmicutes;Streptococcus_cristatus	Saliva	B1s-B2s	0.023496147	0.12254853
Firmicutes;Streptococcus_intermedius	Saliva	B1s-B2s	0.950334739	1
**Firmicutes;Streptococcus_peroris**	**Saliva**	**B1s-B2s**	**0.004266185**	**0.045392206**
Firmicutes;Streptococcus_porcinus	Saliva	B1s-B2s	0.038414324	0.170303502
Firmicutes;Streptococcus_pseudoporcinus	Saliva	B1s-B2s	0.557807611	0.848314496
Firmicutes;Streptococcus_sp._oral_taxon_056	Saliva	B1s-B2s	0.712965125	0.953251607
**Firmicutes;Streptococcus_sp._SK643**	**Saliva**	**B1s-B2s**	**8.34E-04**	**0.015619314**
Firmicutes;Streptococcus_suis	Saliva	B1s-B2s	0.141154012	0.371752149
Firmicutes;Streptococcus_thermophilus	Saliva	B1s-B2s	1	1
Firmicutes;Streptococcus_vestibularis	Saliva	B1s-B2s	0.633325915	0.891347583
Firmicutes;Veillonella_sp._oral_taxon_780	Saliva	B1s-B2s	0.263174191	0.588271721
Proteobacteria;Aggregatibacter_segnis	Saliva	B1s-B2s	0.025086713	0.126670722
**Proteobacteria;Campylobacter_concisus**	**Saliva**	**B1s-B2s**	**5.16E-08**	**1.37E-05**
Proteobacteria;Campylobacter_gracilis	Saliva	B1s-B2s	0.011760504	0.079385837
**Proteobacteria;Campylobacter_showae**	**Saliva**	**B1s-B2s**	**0.001789949**	**0.029008045**
Proteobacteria;Haemophilus_parainfluenzae	Saliva	B1s-B2s	0.006568052	0.058681444
Proteobacteria;Haemophilus_sp._oral_taxon_851	Saliva	B1s-B2s	0.00599101	0.058681444
**Proteobacteria;Haemophilus_sputorum**	**Saliva**	**B1s-B2s**	**3.51E-06**	**4.66E-04**
Proteobacteria;Lautropia_mirabilis	Saliva	B1s-B2s	0.044229415	0.186746419
**Proteobacteria;Neisseria_elongata**	**Saliva**	**B1s-B2s**	**8.40E-04**	**0.015619314**
Proteobacteria;Ralstonia_sp._5_7_47FAA	Saliva	B1s-B2s	0.044014389	0.186746419
Proteobacteria;Rhodanobacter_sp._115	Saliva	B1s-B2s	0.405816604	0.711511435
Saccharibacteria;TM7	Saliva	B1s-B2s	0.006451566	0.058681444
Spirochaetes;Treponema_vincentii	Saliva	B1s-B2s	0.015395201	0.095235429
Actinobacteria;Actinomyces_sp._oral_taxon_849	Saliva	B2s-B2Xs	0.512486352	0.816295627
Actinobacteria;Actinomyces_urogenitalis	Saliva	B2s-B2Xs	0.473869552	0.782914911
Actinobacteria;Rothia_mucilaginosa	Saliva	B2s-B2Xs	0.029496475	0.140108255
Bacteroidetes;Prevotella_maculosa	Saliva	B2s-B2Xs	0.833499632	0.997926198
Bacteroidetes;Prevotella_oris	Saliva	B2s-B2Xs	0.319290549	0.644299985
Firmicutes;Dialister_invisus	Saliva	B2s-B2Xs	0.90092763	1
Firmicutes;Dialister_succinatiphilus	Saliva	B2s-B2Xs	0.885475367	1
Firmicutes;Enterococcus_italicus	Saliva	B2s-B2Xs	0.724121124	0.953545638
Firmicutes;Gemella_sanguinis	Saliva	B2s-B2Xs	0.366875192	0.676841283
Firmicutes;Granulicatella_adiacens	Saliva	B2s-B2Xs	0.967417298	1
Firmicutes;Oribacterium_sinus	Saliva	B2s-B2Xs	0.712965125	0.953251607
Firmicutes;Oribacterium_sp._ACB7	Saliva	B2s-B2Xs	0.504247169	0.810766498
Firmicutes;Selenomonas_sp._oral_taxon_149	Saliva	B2s-B2Xs	0.870194456	1
Firmicutes;Selenomonas_sputigena	Saliva	B2s-B2Xs	0.743702065	0.964998778
Firmicutes;Streptococcus_cristatus	Saliva	B2s-B2Xs	0.9348872	1
Firmicutes;Streptococcus_intermedius	Saliva	B2s-B2Xs	0.118401403	0.342334492
Firmicutes;Streptococcus_peroris	Saliva	B2s-B2Xs	0.53929854	0.841441454
Firmicutes;Streptococcus_porcinus	Saliva	B2s-B2Xs	0.079725196	0.275414312
Firmicutes;Streptococcus_pseudoporcinus	Saliva	B2s-B2Xs	0.961707797	1
Firmicutes;Streptococcus_sp._oral_taxon_056	Saliva	B2s-B2Xs	0.124692525	0.345929986
Firmicutes;Streptococcus_sp._SK643	Saliva	B2s-B2Xs	0.366875192	0.676841283
Firmicutes;Streptococcus_suis	Saliva	B2s-B2Xs	0.329416534	0.644299985
Firmicutes;Streptococcus_thermophilus	Saliva	B2s-B2Xs	0.389422616	0.695210844
Firmicutes;Streptococcus_vestibularis	Saliva	B2s-B2Xs	0.455101207	0.766183044
Firmicutes;Veillonella_sp._oral_taxon_780	Saliva	B2s-B2Xs	0.878026982	1
Proteobacteria;Aggregatibacter_segnis	Saliva	B2s-B2Xs	0.774840302	0.985855076
Proteobacteria;Campylobacter_concisus	Saliva	B2s-B2Xs	0.324548175	0.644299985
Proteobacteria;Campylobacter_gracilis	Saliva	B2s-B2Xs	0.90092763	1
Proteobacteria;Campylobacter_showae	Saliva	B2s-B2Xs	0.803376862	0.988409148
Proteobacteria;Haemophilus_parainfluenzae	Saliva	B2s-B2Xs	1	1
Proteobacteria;Haemophilus_sp._oral_taxon_851	Saliva	B2s-B2Xs	0.966900055	1
Proteobacteria;Haemophilus_sputorum	Saliva	B2s-B2Xs	0.9348872	1
Proteobacteria;Lautropia_mirabilis	Saliva	B2s-B2Xs	0.406577963	0.711511435
Proteobacteria;Neisseria_elongata	Saliva	B2s-B2Xs	0.191313092	0.48008757
Proteobacteria;Ralstonia_sp._5_7_47FAA	Saliva	B2s-B2Xs	0.120758587	0.34539553
**Proteobacteria;Rhodanobacter_sp._115**	**Saliva**	**B2s-B2Xs**	**0.003625355**	**0.041928016**
Saccharibacteria;TM7	Saliva	B2s-B2Xs	0.785497119	0.985855076
Spirochaetes;Treponema_vincentii	Saliva	B2s-B2Xs	1	1

p < 0.05 are highlighted in bold.

**Table A2.** Correlation of microbiome taxa abundance between plaque and saliva samples. The correlation between the taxa is computed based on the abundance at the genus level.

Condition	Correlation *r*	*p* value
A1	0.5241832	1.8980e-14
A1X	0.3467439	1.3307e-06
A2	0.4439990	2.4459e-10
A2S	0.4068658	9.0816e-09
B1	0.5370703	3.2477e-15
B1S	0.6312785	5.8299e-22
B2	0.3608606	4.5107e-07
B2X	0.5017433	3.4545e-13
